# The Army Medical Service

**Published:** 1897-04-10

**Authors:** 


### The Hospital

A Journal of

The Medical Sciences and Hospital Administration.

Vol. XXII.—No. 550.] [April 10, 1897.

### THE ARMY MEDICAL SERVICE.

The condition and prospects of the Medical Department of the Army have for some years been sources of anxiety and regret to all who are acquainted with the facts of the case, and who are able to realise the extent of the disasters which, in time of war, the inefficiency of this branch of the service might entail upon the country. The dissatisfaction of the profession with the existing state of things has reached a stage in which it finds articulate expression in the principal medical schools, so that students of the better class are dissuaded by their teachers from offering themselves as candidates for a military career. The quarrel between medical and combatant officers, or, more correctly, between medical officers and the red tape of the War Office, has now become a chronic one; and, in the public interest, it is high time that some efficient steps should be taken for the purpose of removing the causes in which it has originated and by which it has been sustained.

As a single effort in this direction, a report has just been issued by a body somewhat cumbrously described as a sub-committee of the Parliamentary Bills Committee of the British Medical Association; and this report is well deserving of careful consideration. The *crux* of the whole question seems to be the unreality of the military rank nominally conferred upon the officers of the department. The report points out that the chief medical officer of an army corps of from 40,000 to 50,000 men should be in supreme command, under the sanctions of military discipline and authority of the 2,000 or more men whose duties embrace the care of the sick and wounded, or the general control of ambulances, hospitals, and their attendant appliances; and that he should also, preferably by holding a position on the Head-quarter Staff, be sufficiently acquainted with the intentions and dispositions of the General to be prepared for all probable contingencies, and to have his force in hand and on the spot when it is wanted, so as promptly to clear the front of wounded and ineffectives. The authors of the report deem it essential to the usefulness, and hence to the popularity of the service, that it should be constituted, like the Royal Engineers or the Royal Artillery, with simple military titles to which the name of the corps should be added, as Captain or Major A., Medical Staff Corps; and they think that, if this were conceded, and the military rank rendered a reality in relation to boards and committees, the remaining grievances, themselves mainly results of the now prolonged agitation, might without much difficulty be removed. They express a firm conviction that nothing else will suffice, and that, if military rank be still withheld, another great war would witness a complete breakdown of the whole of our army medical service.

The argument of the War Office has always been that they want doctors and not soldiers; and they treat the desire for military rank as if it were a mere expression of the wish of medical jackdaws to strut in borrowed plumes, instead of being, as it is, a just demand for sufficient authority for the proper discharge of onerous duties. The War Office may have some show of reason for its contention. Its treatment of the service has compelled it to go into the highways and hedges for recruits; and it is quite possible that inferior medical students, who have scrambled through an examination after many rejections, will think more of their uniforms than of the welfare of the men committed to their charge. The way to avoid this is not to refuse what is necessary, but to render the conditions of the service such as will attract competent and self-respecting men. A man who is not a good doctor may possibly find refuge from his consciousness of professional insignificance by fancying himself a soldier; but a man who is a good doctor is likely to be quite content with due recognition of his merits in that capacity. A minor matter, but one of some importance, is the destruction of the tie which formerly bound a surgeon to his regiment, which became his home and family, and the home and family of his wife if he married. Now, as an unattached member of a department, he has neither home nor brother officers; and his wife is equally external to any distinctively military circle. The report proposes that members of the Medical Staff Corps should be attached to regiments for terms of years; or, in other words, that there should be a sufficient return to the old system to yield its social advantages to all concerned. We fancy that the imagination of the average official will not be unduly oppressed by a document which emanates from an unnamed section of a great unknown; but, in spite of this disadvantage, the report itself is eminently reasonable, has evidently been prepared under the guidance of complete knowledge of the questions to which it refers, and is entirely worthy of the attention of the public and of Parliament. The worst feature of a "strong" Government is that it can neither be coerced or cajoled into the institution of reforms which would be troublesome to its permanent officials, and therefore we are not sanguine enough to expect any immediate change. But the condition of the Medical Department seems likely to go steadily from bad to worse; and we trust that ultimately, in the language of the transpontine drama, "a time will come."

